# Ethanol-Induced Alterations of T Cells and Cytokines after Surgery in a Murine Infection Model

**DOI:** 10.1155/2017/1067598

**Published:** 2017-11-20

**Authors:** Nadine Lanzke, Mario Menk, Clarissa von Haefen, Lilit Sargsyan, Bianca Scharf, Klaus-Dieter Wernecke, Claudia D. Spies

**Affiliations:** ^1^Department of Anesthesiology and Intensive Care Medicine, Campus Charité Mitte and Charité Campus Virchow-Klinikum, Charité-Universitätsmedizin Berlin, Augustenburger Platz 1, 13353 Berlin, Germany; ^2^SOSTANA GmbH, Wildensteiner Straße 27, 10318 Berlin, Germany

## Abstract

**Background:**

Interactions between alcohol, infection, and surgery and their effect on differentiation and functionality of T helper cells are not yet completely understood. We hypothesized that alcohol and surgery disturb differentiation of T helper cells and contribute to an impaired immune response.

**Methods:**

Mice were treated with alcohol for two weeks. Saline treatment served as control. Clinical performance and weight were assessed. On day 14, a median laparotomy was performed and animals were challenged with* Klebsiella pneumoniae *intranasally. Bacterial load was determined in lungs and blood. T helper cell subpopulations and the released cytokines were assessed in lungs, spleens, and plasma. Key transcription factors of T cell differentiation were evaluated.

**Results:**

Alcohol significantly impaired clinical appearance and body weight of animals with postsurgical infection (*p* < 0.05). Bacterial load was significantly higher after alcohol treatment (*p* < 0.05). T helper cell subsets and released cytokine levels were significantly altered in lung, but not in spleen. Expression of transcription factors of T helper cell lineage commitment did not translate into different counts of T helper cells.

**Conclusions:**

Alcohol and surgery lead to significant cellular and functional modulations of T helper cells during postsurgical infection. These effects may contribute to an impaired immune response after surgery.

## 1. Introduction

Acute and chronic alcohol abuse have been shown to impair cellular immunity by provoking significant changes in T cell numbers and their activity [1–4]. CD3^+^ T cells are inhibited in their proliferation and the numbers of CD4^+^ and CD8^+^ T cells are significantly decreased. Alcohol consumption, which is one of the leading risk factors for morbidity and mortality worldwide [5], leads to lymphocytopenia [6] as well as leucocytopenia [7]. In surgical units the prevalence of patients with a history of alcohol abuse is about 20% [8]. These patients exhibit a three- to fivefold increase of complications like sepsis or nosocomial infections after surgery. This has been shown to be associated with a deranged preoperative ratio of T helper cells [9]. Surgical procedure likewise causes immunosuppression by impacting T cells [10, 11] and thereby increases susceptibility to infection [12]. Already one day after surgery decreased T cell functions have been observed [13]. Further, T lymphocyte proliferation and cytokine secretion are severely impaired after major surgery [14, 15]. T cells and their distinct subpopulations, that is, Th1, Th2, T helper 17 (Th17) cells, and regulatory T cells (Treg cells), have a central role during the postsurgical immune response [16–19].

However, only few experimental models depict the clinical situation, involving alcohol administration, a surgical procedure, and the exposure to an infectious agent. In an earlier work, we used a model with 8 days of alcohol exposition and focused on the effects of ethanol on cytokine production in an infection scenario after surgery. In order to investigate the effects of alcohol in an operative setting and eventually to assess the overall situation as seen in patients in operative units, we choose this three-hit model aiming at the clinical circumstances. We found that alcohol intake prior to surgery worsened clinical appearance of affected animals, led to a more severe pulmonary infection with* K. pneumoniae*, and was associated with more tissue destruction and increased levels of IL-6 [20]. Given this previous result, we aimed to intensify the alcohol-induced impairment of immunity and to test the hypothesis that several (more) days of excess alcohol consumption results in a more pronounced immunosuppression with respect to T helper cells and cytokine production. The putative combined effect of alcohol and surgery might disturb both, proper differentiation and functionality of T helper cell subpopulations and might—at least in part—contribute to an impaired immune response after surgery. Therefore, the aim of our study was to investigate T helper cell subpopulations in the clinically highly relevant setting of alcohol exposure, surgical stress, and pulmonary infection.

## 2. Materials and Methods

### 2.1. Animals

Nine-week-old female Balb/c mice (specific-pathogen-free), obtained from Charles River (Sulzfeld, Germany), were used in all experiments. Food and water were allowed ad libitum. Animals were kept on a 12 h/12 h light/dark cycle in a temperature controlled and specific-pathogen-free environment within the animal care facility at the Campus Virchow-Klinikum, Charité-Universitätsmedizin Berlin. All experimental procedures were approved by the Committee on Use and Care of Animals at the Charité-Universitätsmedizin Berlin, Germany, by the state animal committee (LaGeSo, Germany; approval number G0213/05). The protocols were performed in accordance with the National Institutes of Health ‘‘Guidelines for the Care and Use of Laboratory Animals.”

### 2.2. Group Assignment

In a randomized design, 8 groups with 8 mice each were set up: one-half of mice were treated 14 days with ethanol, and the other half received saline. On day 14 of experiment all groups underwent surgery and were subjected to a midline laparotomy under sterile conditions and general, inhalative anesthesia as described previously [20]. Two days after surgery four of the eight groups (ethanol- and saline-treated) were given* K. pneumoniae*. The four other groups (ethanol- and saline-treated) were given saline as sham infection. Mice were sacrificed 24 or 48 hours after* K. pneumoniae* application and organs and blood were harvested.

### 2.3. Alcohol Treatment

After 8 days of acclimatization, mice were pretreated daily with intraperitoneal ethanol at a dose of 3.8 mg/kg body weight for 14 days. Concentration of ethanol i.p. was 20%. Ethanol was given intraperitoneally to ensure the same weight adapted dose for all mice. Both, oral and intraperitoneal gavage, are methods of systemic administration. We chose a duration of 14 days to provide sufficient time for the development of immunological complications due to alcohol exposure on one hand, but to prevent the event of organ complications (e.g., liver damage) as seen after longtime chronic alcohol treatment. As the duration of alcohol treatment does not correspond with chronic alcohol exposition neither dependency nor withdrawal was expected [21]. Control groups received saline similarly.

### 2.4. Surgical Intervention

The surgical intervention in respective groups was performed after administration of the final alcohol dose on day 14 of experiment. A midline laparotomy was performed under sterile conditions and general, inhalative anesthesia as described previously [20].

### 2.5. *Klebsiella pneumoniae* Inoculation

*Klebsiella pneumoniae* strain 43816 (ATCC, Rockville, MD) Trevisan Serotype 2 was used. On day 16 of the experiment, mice of infection-groups were anesthetized with ketamine (100 mg/kg body weight) and midazolam (4 mg/kg body weight). 1 × 10^4^ colony forming units (CFUs) of* K. pneumoniae* were administered intranasally using a pipette in a total volume of 50 *μ*l saline as described elsewhere [22]. In order to control the concentration of bacteria, an aliquot was plated on blood agar and incubated for 24 hours at 37°C and CFUs were determined for each experiment. Mice of the control groups were likewise anesthetized and received 50 *μ*l of sterile saline intranasally using a pipette.

### 2.6. Clinical Rating

Clinical condition and body weight of animals were evaluated on each day of the experiment on the basis of clinical signs. Motor activity, mucous membranes, and piloerection as a sign for grooming were assessed. For each item a score from zero to four points was given. Zero points were assigned for no clinical impairment, one point was for mild, two points were for moderate, three points were for moderate to severe, and four points were for very severe impairment of the respective item. Clinical rating score was evaluated for each group.

### 2.7. Extraction of Organs

**Figure 1:**
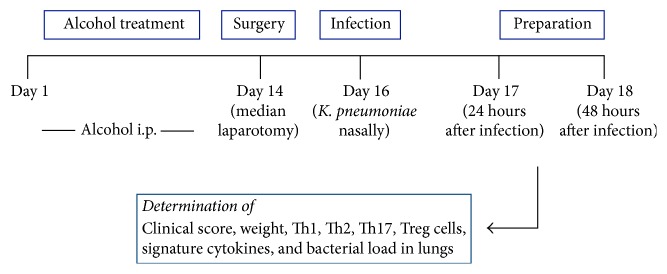
Study protocol.

Mice were sacrificed 24 hours or 48 hours after* K. pneumoniae* inoculation, respectively. An abdominal laparotomy and a median thoracotomy were performed. Lungs, spleens, and blood plasma samples were harvested and stored at −80°C for further investigation. The study protocol is shown in Figure 1.

### 2.8. Flow Cytometric Analysis of T Cell Subsets

After organ removal, lungs were aseptically minced and mechanically homogenized. In short, lung lymphocytes were obtained by preparing single-cell suspensions from the organs. Erythrocytes were removed using erylysis buffer (Buffer EL Erythrocyte lysis, Qiagen GmbH, Hilden). Cells were suspended in PB buffer (0,2% PBS-BSA, Dulbecco's Phosphat Buffered Saline without Ca and Mg: PAA Laboratories GmbH, Austria, Albumin Serum, Sigma Aldrich, Steinheim, Germany) and stained with trypan blue (Sigma, France) to adjust a cell count of 10 × 10^6^/ml. Cells were stimulated in RPMI with phorbol 12-myristate 13-acetate (PMA) (Sigma Aldrich, Steinheim, Germany) 5 ng/ml per 2 × 10^6^ cells and ionomycin (1 *μ*g/ml) (Sigma Aldrich, Steinheim, Germany) and incubated for 2 hours in a humidified air incubator at 37°C, 5% CO_2_. Golgi-Stop (Protein transport inhibitor, BD Biosciences, Germany) was added at a final concentration of 5 *μ*g/ml and cells were incubated again for 2 hours.

Cultured cells were washed with PBS-buffer and incubated with Fc-blocking solution (Purified Rat Anti-Mouse CD16/CD32, Mouse BD Fc Block, BD Bioscience, 2.4 G2) preventing nonspecific antibody binding. Afterwards, cells were stained and incubated with CD3 Pacific Blue (Pacific Blue Hamster Anti-Mouse CD3, BD Biosciences, 500A2), CD4 APC-H7 (APC-H7 Rat Anti-Mouse CD4, BD Biosciences, GK1.5), and CD8 PerCP (PerCP Rat Anti-Mouse CD8a, BD Biosciences, 53-6.7) before fixation with Perm-Fix-buffer (Cytofix/Cytoperm Solution for fixation and permeabilization, BD Biosciences) for 10 minutes. Isotype Controls, Pacific Blue Hamster IgG2, *κ* Isotype Control (BD Bioscience, B81-3), APC-H7 Rat IgG2b, *κ* Isotype Control (BD Bioscience, R35-95), and PerCP Rat IgG2a, *κ* Isotype Control (BD Bioscience, R35-95), were used at the same concentrations as the respective anti-cytokine antibodies.

For intracellular staining, cells were washed and then permeabilized with Perm Wash Buffer (BD, Biosciences) and incubated with IFN-*γ* FITC (FITC Rat Anti-Mouse IFN*γ*, BD Bioscience XMG1.2), IL-4 APC (APC Rat Anti-Mouse IL-4, BD Bioscience, 11B11), IL-17 PE (PE Rat Anti-Mouse IL-17, BD Bioscience, TC11-18H10), and Foxp3 PE-Cy7 (Phycoerythrin-Cy7 (PE-Cy7) Anti-Mouse/Rat Foxp3, eBioscience, FJK-16s). Isotype Controls Iso FITC (FITC Rat IgG1, *κ* Isotype Control, BD Bioscience, R3-34), Iso APC (APC Rat IgG1, *κ* Isotype Control, BD Bioscience, R3-34), Iso PE (PE Rat IgG1, *κ* Isotype Control, BD Bioscience, R3-34), and Iso PE-Cy7 (Phycoerythrin-Cy7 (PE-Cy7) Rat IgG2a Isotype Control, eBioscience, eBR2a) were used at the same concentrations as the respective anti-cytokine antibodies.

Cells were washed with PBS and samples were analyzed on a FACS-Canto-II flow cytometer (Becton-Dickinson, Heidelberg, Germany). Analysis was performed with FACSDiva Software. Data are given as number of cell counts per 50.000 lymphocytes and presented as means +/− standard deviation.

### 2.9. Cytokine Detection

Concentrations of IL-6, IL-4, IFN*γ*, TNF-*α*, IL-17, and IL-10 in lungs of animals were evaluated using the commercial mice/rat cytometric bead array (Becton-Dickinson, Heidelberg, Germany) for flow cytometric analysis. In short, 50 *μ*l of protein lysate of organs or plasma aliquots were used per sample following the manufacturer's instructions. Sensitivity of this system was between 1.9 and 7.2 pg/ml for each of the respective cytokines. Protein concentration of lysates was determined using the bicinchoninic acid assay (Pierce, Rockford, USA). Data are expressed as *μ*g/mg of organ protein or pg/ml plasma, respectively.

### 2.10. Quantitative RT-PCR

Total RNA was isolated using the Absolutely RNA kit (Stratagene, La Jolla, USA) according to the manufacturer's protocol including a DNase digest. cDNA synthesis was performed using random hexamer primers and M-MLV reverse transcriptase (RNase H minus; Promega, Mannheim, Germany); no template controls (NTCs) and reactions without addition of reverse transcriptase (RT−) served as negative controls. cDNA was quantified by real-time PCR using FAM/TAMRA labeled specific probes (provided by Applied Biosystems, Darmstadt, Germany). Data represent the mean expression level ± standard deviation (standardized to hypoxanthine guanine phosphoribosyltransferase (HPRT) expression) calculated according to the 2^−ΔΔCT^ method of at least three independent measurements per cDNA (technical triplicates).

### 2.11. Determination of Colony Forming Units (CFUs)

After mixing, blood was plated on Columbia blood agar (Oxoid, Cambridge, UK) and incubated at 37°C for 24 hours and colony forming units of* K. pneumoniae* were determined. After removal of lungs they were minced and a serial dilution series was plated on blood agar and incubated for 24 hours at 37°C. Colonies were counted and CFUs per ml blood or CFUs per g lung tissue were calculated.

### 2.12. Statistical Analysis

Statistical analysis was performed with nonparametric tests (Mann–Whitney* U* test). Numerical calculations were carried out with the GraphPad Prism Software (GraphPad, La Jolla, USA). A *p* value of less than 0.05 was considered statistically significant. In the figures, all data are expressed as mean +/− standard deviations or mean +/− standard error of the mean (SEM) unless otherwise stated. For more clarity, statistically significant differences as a result of ethanol pretreatment are marked with asterisks (*∗*), significant differences due to the infection are marked with dollar signs ($), and significant differences over course of time, but in otherwise identically treated groups, are marked with hash keys (#).

## 3. Results

### 3.1. Basic Animal Characteristics, Body Weight, and Clinical Rating

**Figure 2:**
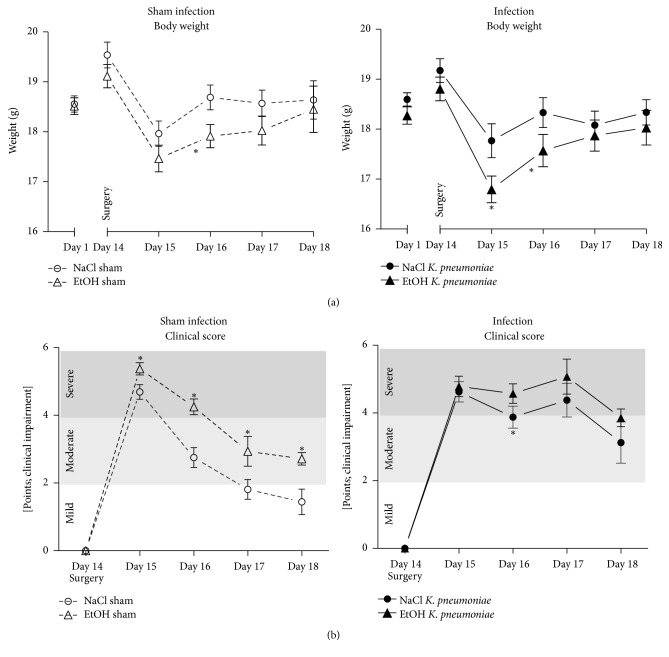
Analysis of body weight and clinical score in a murine model of ethanol exposition for 2 weeks, surgery, and pulmonary infection. (a) Development of body weight in sham-infected animals (left panel) and in animals with pulmonary infection with* Klebsiella pneumoniae* (right panel) over course of time in ethanol-treated (black/white triangle) and respective saline-treated controls (black circles/white circles). ^*∗*^*p* < 0.05 when compared to time-matched control group; data are presented as mean ± SEM, Mann–Whitney* U* test (*n* = 8 each group). (b) Development of clinical score in sham-infected animals (left panel) and in animals with pulmonary infection with* Klebsiella pneumoniae* (right panel) over course of time in ethanol-treated (black/white triangle) and respective saline-treated controls (black circles/white circles). For clinical scoring, motor activity, mucous membranes, and piloerection were assessed. For each item a score from zero to four points was given (0 points = no clinical impairment, 1 point = mild, 2 points = moderate, 3 points = moderate to severe, and 4 points = very severe impairment of the respective item). Clinical rating score was evaluated for each group. ^*∗*^*p* < 0.05 when compared to time-matched control group; data are presented as mean ± SEM, Mann–Whitney* U* test (*n* = 8 each group). ETOH: ethanol treatment; NaCl: saline-treated control;* K*.* pneumoniae*: pulmonary infection with* Klebsiella pneumoniae*.

Basic animal characteristics such as body weight or clinical scoring did not differ significantly between groups on first day of the experiment (Figure 2). After surgery, body weight decreased significantly in both, saline- and ethanol-treated mice, and slowly returned to presurgical values. Body weight of ethanol-treated mice did recover more slowly. This effect reached statistical significance on day 16 of the experiment (Figure 2(a), left). In mice with pulmonary infection with* K. pneumoniae*, the decrease of body weight after surgery was even more pronounced with a history of ethanol pretreatment compared to saline-treated mice (Figure 2(a), right). In both cases, that is, with and without pulmonary infection, pretreatment with ethanol caused a prolonged impairment of body weight after surgical stress.

Clinical rating of animals was significantly impaired after surgery in all groups (Figure 2(b)). Motor activity, appearance of mucous membranes, and piloerection were severely impaired after surgery in saline-treated mice but rapidly recovered to normal conditions. Throughout the whole observation period, ethanol-treated mice showed a significantly impaired physical status and recovered more slowly after surgical intervention (Figure 2(b), left panel). In mice with pulmonary infection, clinical status remained severely impaired after surgery (Figure 2(b), right). In any case, pretreatment with ethanol caused a worsened clinical condition indicating a compromised physical well-being of the animals.

### 3.2. Bacterial Load after Pulmonary Infection

**Figure 3:**
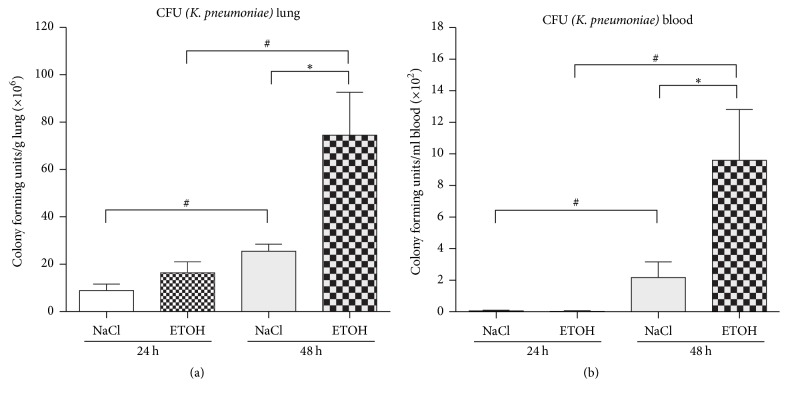
Analysis of colony forming units (CFUs) of* Klebsiella pneumoniae* in a murine model of ethanol exposition for 2 weeks, surgery, and pulmonary infection. (a) Colony forming units (CFU x/g lung tissue × 10^6^) in the lung of animals in ethanol-treated (ETOH) and saline-treated controls (NaCl) at 24 hours or 48 hours after pulmonary infection. ^#^*p* < 0.05 when comparing 24 hours to 48 hours (time-dependent effects); ^*∗*^*p* < 0.05 when comparing ethanol treatment and saline-controls at the same time-point (ethanol-dependent effects); data are presented as mean ± SEM, Mann–Whitney* U* test (*n* = 8 each group). (b) Colony forming units (CFU x/ml blood × 10^2^) in the blood of animals in ethanol-treated (ETOH) and saline-treated controls (NaCl) at 24 hours or 48 hours after pulmonary infection. ^#^*p* < 0.05 when comparing 24 hours to 48 hours (time-dependent effects); ^*∗*^*p* < 0.05 when comparing ethanol treatment and saline-controls at same time-point (ethanol-dependent effects); data are presented as mean ± SEM, Mann–Whitney* U* test (*n* = 8 each group). CFU: colony forming unit; ETOH: ethanol treatment; NaCl: saline-treated control;* K. pneumoniae*:* Klebsiella pneumoniae*.

Inoculation of* K. pneumoniae* caused considerable levels of CFUs in the lungs 24 hours after infection (Figure 3(a)). CFUs were significantly increased at 48 hours compared to 24 hours in saline-treated (^#^*p* = 0.003) and ethanol-treated mice (^#^*p* = 0.005). Moreover, ethanol treatment led to a significant increase of CFUs at 48 hours (^*∗*^*p* = 0.014) when compared to saline-treated controls. In the blood, ethanol treatment caused a significant increase of CFUs at 48 hours (^*∗*^*p* = 0.0029) after infection (Figure 3(b)). These results indicate an impaired pulmonary clearance of bacteria and a more severe status of pulmonary infection in the clinical course after ethanol treatment.

### 3.3. Effects on CD3^+^, CD4^+^, and CD8^+^ Cells in the Lung and Spleen

**Figure 4:**
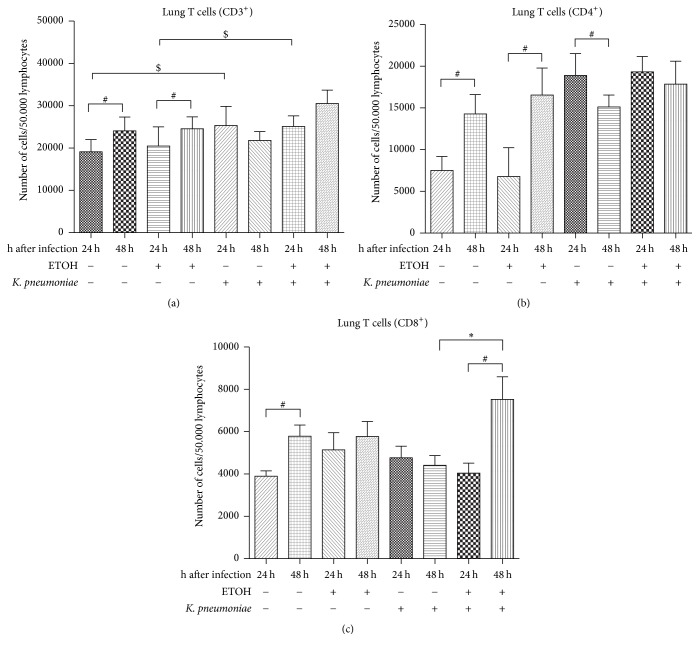
Flow cytometric analysis of lung and splenic T lymphocyte subsets in a murine model of ethanol exposition for 2 weeks, surgery, and pulmonary infection. (a) Flow cytometric analysis of CD3+ cells in the lung in ethanol-treated (ETOH) and saline-treated controls (NaCl) at 24 hours or 48 hours after pulmonary infection with* Klebsiella pneumoniae*. (b) Flow cytometric analysis of CD4+ cells in the lung in ethanol-treated (ETOH) and saline-treated controls (NaCl) at 24 hours or 48 hours after pulmonary infection with* Klebsiella pneumoniae*. (c) Flow cytometric analysis of CD8^+^ cells in the lung in ethanol-treated (ETOH) and saline-treated controls (NaCl) at 24 hours or 48 hours after pulmonary infection with* Klebsiella pneumoniae*. All cell counts are given as number of cells per 50.000 lymphocytes. ^*∗*^*p* < 0.05 when comparing ethanol treatment and saline-treated controls at the same time-point (ethanol-dependent effects); ^$^*p* < 0.05 when compared to time- and treatment-matched control (infection-dependent effects); ^#^*p* < 0.05 when comparing 24 hours to 48 hours (time-dependent effects); data are presented as mean ± SEM, Mann–Whitney* U* test (*n* = 8 each group). CD: cluster of differentiation; ETOH: ethanol treatment;* K*. pneumoniae: pulmonary infection with* Klebsiella pneumoniae*.

To get a general overview on CD3^+^ T cell subsets, we analyzed respective populations by flow cytometric analysis. At 24 hours after infection, lung CD3^+^ cells were significantly increased as a consequence of infection with* K. pneumoniae* in saline- (^$^*p* = 0.007) and ethanol-treated mice (^$^*p* = 0.029). Also, ethanol and infection combined led to a significant increase of CD3^+^ cells compared to saline treatment with sham infection (*p* = 0.0022) at both, 24 hours and 48 hours. Both saline-treated/sham-infected (^#^*p* = 0.0104) and ethanol-treated/sham-infected (^#^*p* = 0.049) mice showed a significant rise of CD3^+^ cells in their lungs at 48 hours compared to 24 hours (Figure 4(a)). In the spleen, overall count of CD3^+^ cells did not differ significantly due to any intervention (not shown).

CD3^+^/CD4^+^ T helper cells did significantly increase without infection over course of time after surgery but did significantly decrease in a postsurgical infection scenario (Figure 4(b)). There was no statistical significant effect due to ethanol pretreatment regarding CD4^+^ T helper cells in the respective groups. In the spleen, overall count of CD4^+^ cells did not differ significantly due to any intervention or at any considered time (not shown).

Cytotoxic CD3^+^/CD8^+^ T cells did significantly increase over course of time after surgery in lungs of saline-treated/sham-infected mice (Figure 4(c)). This effect, however, was not detectable with a history of ethanol treatment. After pulmonary infection with* K. pneumoniae, *cytotoxic CD8^+^ T cells did significantly increase at 48 hours in ethanol-treated, but not in saline-treated mice. Splenic CD3^+^/CD8^+^ cells did not differ significantly due to any intervention or at any considered time (not shown).

### 3.4. Effects on Th1 Cells, Transcription of* t-bet*, and IFN*γ* Cytokine Release in the Lung

**Figure 5:**
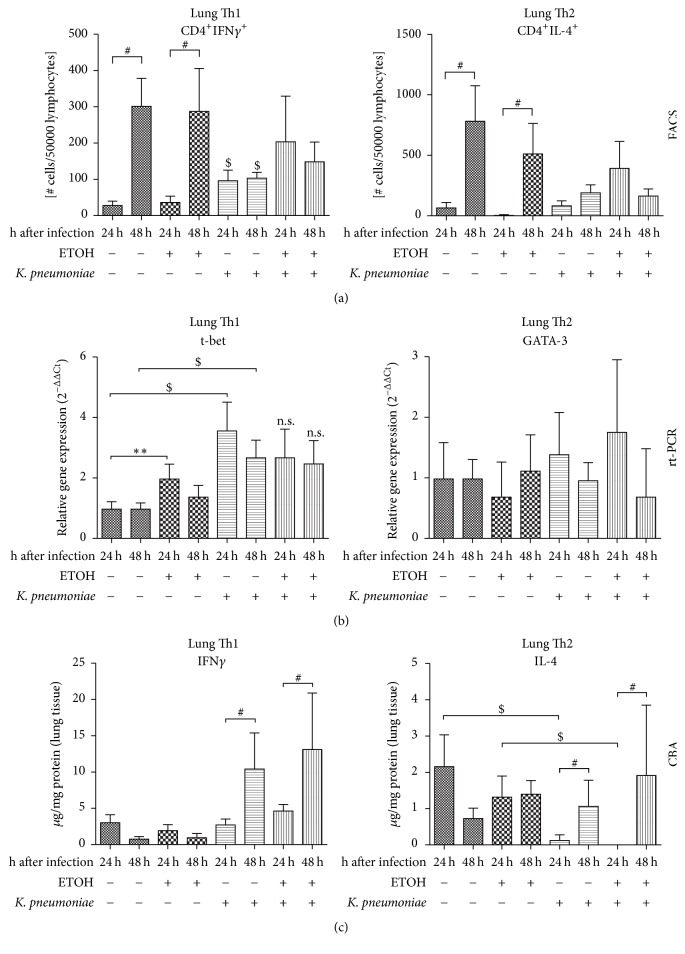
Flow cytometric analysis of indicated T helper cell subsets in the lung, relative gene expression of key transcription factors, and release of cytokine protein in a murine model of ethanol exposition for 2 weeks, surgery, and pulmonary infection. (a) Flow cytometric analysis of CD4^+^/IFN-*γ*^+^ cells (left) and CD4^+^/IL-4^+^ cells (right) in the lung of ethanol-treated (ETOH) and saline-treated controls (NaCl) at 24 hours or 48 hours after pulmonary infection with* Klebsiella pneumoniae*. Cell counts are given as number of cells per 50.000 lymphocytes. (b) Transcriptional regulation of indicated transcription factors assessed by real-time polymerase chain reaction. Expression of *β*-actin served as housekeeping gene; expression of controls was set to 100%. (c) Protein levels of indicated cytokines were analyzed by cytometric bead array (CBA) and given as *μ*g/mg protein of lung tissue. ^*∗∗*^*p* < 0.001 when comparing ethanol treatment and saline-treated controls at the same time-point (ethanol-dependent effects); ^$^*p* < 0.05 when compared to time- and treatment-matched control (infection-dependent effects); ^#^*p* < 0.05 when comparing 24 hours to 48 hours (time-dependent effects); data are presented as mean ± SEM, Mann–Whitney* U* test (*n* = 8 each group). CD: cluster of differentiation;* t-bet*: T-box transcription factor;* GATA-3*: trans-acting T-cell-specific transcription factor GATA-3; IFN-*γ*: interferon gamma; IL: interleukin; ETOH: ethanol treatment;* K*.* pneumoniae*: pulmonary infection with* Klebsiella pneumoniae*.

CD4^+^/IFN*γ*^+^ cells did significantly increase in the lungs over course of time (Figure 5(a), left). The overall count of Th1 cells did significantly increase in infected animals, but the postsurgical increase of Th1 cells at 48 hours was significantly hampered in an infection scenario (*p* = 0.007). Again, this effect occurred independently of ethanol pretreatment. Taken together, both surgery and infection induced effects on Th1 lineage differentiation.

By trend, ethanol treatment led to an increase of* t-bet *transcription in lungs over course of time after surgery (Figure 5(b), left). This effect reached statistical significance at 24 hours (^*∗∗*^*p* < 0.001), but not at 48 hours. Infection significantly increased* t-bet *transcription in lungs of saline-treated mice at both, 24 and 48 hours, compared to saline-treated control. This effect, that is, the transcriptional upregulation of* t-bet*, was diminished in ethanol-treated animals.

A significant increase of IFN*γ* protein was only detected in infected animals. Surgery alone did not cause profound cytokine release over course of time and regardless of ethanol treatment (Figure 5(c), left). Highest levels of IFN*γ* were found at 48 hours after infection. In summary, ethanol, infection, and surgery had significant impact on cellular differentiation of T helper cells of the Th1 sublineage, transcription levels of* t-bet, *and the released signature cytokine IFN*γ* in the lung.

Analysis of the above Th1-specific parameters in the spleen of respective animals revealed no significant changes in cell count,* t-bet* expression, or IFN*γ* levels (data not shown).

### 3.5. Effects on Th2 Cells, Transcription of GATA-3, and IL-4 Cytokine Release in the Lung

CD4^+^/IL-4^+^ cells did significantly increase in the lungs over course of time after surgical intervention (Figure 5(a), right). By trend, cell counts of Th2 cells were lower in pulmonary infection, but there were no significant effects attributable to previous ethanol treatment. However, the postsurgical increase of Th2 cells over course of time in our model was hampered whenever pulmonary infection was present. Ethanol treatment caused an increase in postsurgical Th2 cell numbers at 24 hours in infected animals by trend, but this effect did not reach statistical significance.

The mRNA levels of the key transcription factor* GATA-3 *did not change significantly due to any intervention or any pretreatment (Figure 5(b), right).

On the protein level, the release of IL-4 was significantly diminished in saline-treated, infected mice indicating a functional inhibition of Th2 cells after infection (Figure 5(c), right). This effect also occurred in ethanol-pretreated animals. In infection, IL-4 levels significantly increased over course of time. In ethanol-treated groups the increase at 48 hours was more intense, but this effect did not reach statistical significance.

The analysis of the abovementioned Th2-specific parameters in the spleen of respective animals revealed no significant changes in Th2 cell count,* GATA-3 *expression, or IL-4 levels (data not shown).

### 3.6. Effects on Th17 Cells, Transcription of* RORγT*, and IL-17 Cytokine Release in the Lung

**Figure 6:**
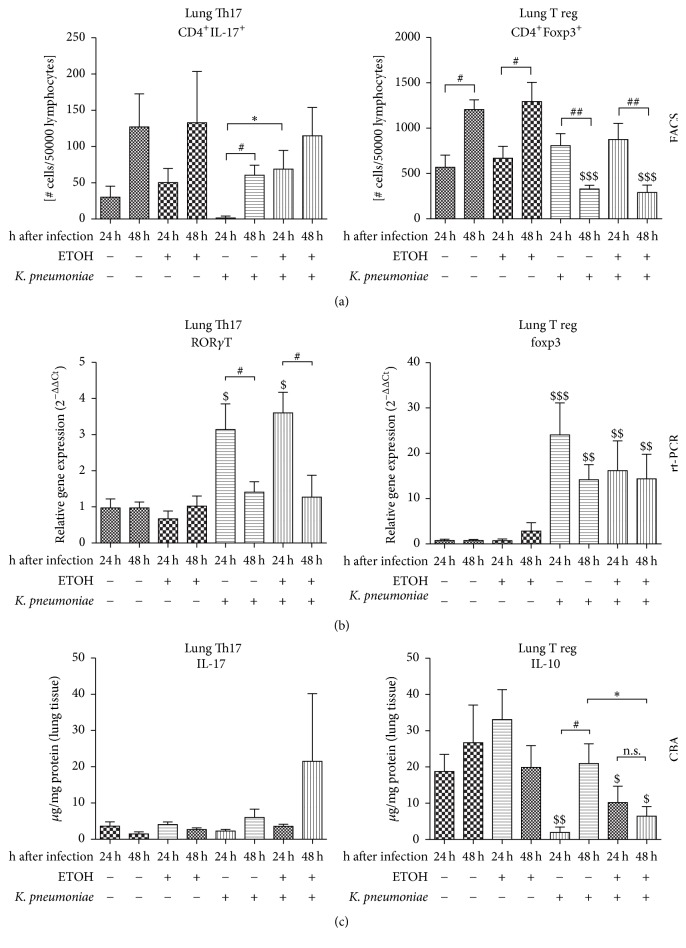
Flow cytometric analysis of indicated T helper cell subsets in the lung, relative gene expression of key transcription factors, and release of cytokine protein in a murine model of ethanol exposition for 2 weeks, surgery, and pulmonary infection. (a) Flow cytometric analysis of CD4^+^/IL-17^+^ cells (left) and CD4^+^/Foxp3^+^ cells (right) in the lung of ethanol-treated (ETOH) and saline-treated controls (NaCl) at 24 hours or 48 hours after pulmonary infection with* Klebsiella pneumoniae*. Cell counts are given as number of cells per 50.000 lymphocytes. (b) Transcriptional regulation of indicated transcription factors assessed by real-time polymerase chain reaction. Expression of *β*-actin served as housekeeping gene; expression of controls was set to 100%. (c) Protein levels of indicated cytokines were analyzed by cytometric bead array (CBA) and given as *μ*g/mg protein of lung tissue. ^*∗*^*p* < 0.05 when comparing ethanol treatment and saline-treated controls at the same time-point (ethanol-dependent effects); ^$^*p* < 0.05; ^$$^*p* < 0.01; ^$$$^*p* < 0.001 when compared to time- and treatment-matched control (infection-dependent effects); ^#^*p* < 0.05; ^##^*p* < 0.01 when comparing 24 hours to 48 hours (time-dependent effects); data are presented as mean ± SEM, Mann–Whitney* U* test (*n* = 8 each group). n.s.: not significant; CD: cluster of differentiation;* RORγT*: retinoid orphan receptor gamma T transcription factor;* foxp3*: forkhead box p3 T-cell-specific transcription factor; IL: interleukin; ETOH: ethanol treatment;* K*.* pneumoniae*: pulmonary infection with* Klebsiella pneumoniae*.

Th17 cells showed an increase over course of time after surgical intervention by trend (Figure 6(a), left). 24 h after pulmonary application of* K. pneumoniae*, lung CD4^+^/IL-17^+^ cells were significantly elevated in ethanol-treated mice compared with controls (^*∗*^*p* = 0.0103). This effect, however, was transient and not detectable anymore at 48 hours. Over course of time, there was a significant increase of Th17 cells in saline-treated mice with infection. Pretreatment with ethanol abolished this effect.

The mRNA expression of the transcription factor* RORγT* did not change significantly over course of time without pulmonary infection (Figure 6(b), left).* RORγT *expression was significantly augmented as a consequence of infection compared to sham infection in saline- (^$^*p* = 0.0079) and ethanol-treated mice (^$^*p* = 0.0003). This effect was transient and only detectable at 24 hours after infection. At 48 hours the increased transcription rate was not detectable anymore. Ethanol treatment did not lead to any significant changes in* RORγT *expression at any considered time-point.

The described changes in Th17 cellular differentiation and expression of* RORγT *did not translate into measurable changes in IL-17 cytokine release (Figure 6(c), left).

The analysis of the abovementioned Th17-specific parameters in the spleen of respective animals revealed no significant changes in Th17 cell count,* RORγT *expression, or IL-17 cytokine levels (data not shown).

### 3.7. Effects on T Regulatory Cells, Transcription of Foxp3, and IL-10 Cytokine Release in the Lung

Treg cells were not significantly altered at 24 hours due to any intervention (Figure 5(a), right panel). By trend, there was an increase of cellular count of this T helper cell subset due to infection. Over course of time, Treg cells significantly increased from 24 hours to 48 hours. Infection itself led to a highly significant decrease in Treg cells when compared with respective controls. This effect occurred only at 48 after infection, but not at 24 hours. Over course of time, there was a significant decrease in number of Treg cells when compared with respective saline- or ethanol-treated controls. There was no effect of previous ethanol treatment.

The expression of* Foxp3* mRNA was only induced when pulmonary infection was present (Figure 6(b), right). Also,* Foxp3* mRNA was significantly augmented in lungs from ethanol-treated infected mice compared to saline-treated sham-infected mice at 24 hours and 48 hours. Ethanol treatment had no significant influence in this regard.

By trend, pulmonary infection with* K. pneumoniae* led to decreased amounts of IL-10 cytokine in the lung (Figure 6(c), right). This effect was only significant at 24 hours in both, ethanol- and saline-treated mice. Ethanol treatment significantly reduced IL-10 cytokine release 48 hours after infection. IL-10 levels were significantly higher in lungs of saline-treated, infected mice at 48 hours compared to 24 hours. This increase of anti-inflammatory IL-10 was abolished with ethanol treatment.

### 3.8. Proinflammatory Cytokines IL-6 and TNF-*α* in Lung Tissue

**Figure 7:**
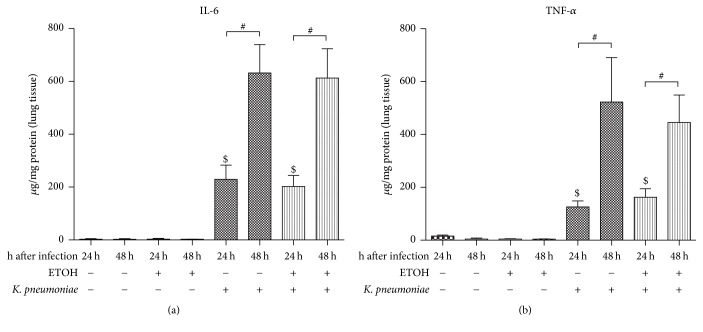
Protein levels of (a) IL-6 and (b) TNF-*α* in lung tissue in a murine model of ethanol exposition for 2 weeks, surgery, and pulmonary infection. Protein levels of indicated cytokines were analyzed by cytometric bead array (CBA) and given as *μ*g/mg protein of lung tissue in the lung of ethanol-treated (ETOH) and saline-treated controls (NaCl) at 24 hours or 48 hours after pulmonary infection with* Klebsiella pneumoniae*. ^$^*p* < 0.05 when compared to time- and treatment-matched control (infection-dependent effects); ^#^*p* < 0.05 when comparing 24 hours to 48 hours (time-dependent effects); data are presented as mean ± SEM, Mann–Whitney* U* test (*n* = 8 each group). IL: interleukin; TNF-*α*: tumor necrosis factor; ETOH: ethanol treatment;* K*.* pneumoniae*: pulmonary infection with* Klebsiella pneumoniae*.

Proinflammatory early response cytokines IL-6 and TNF-*α* were only detectable in a significant amount whenever infection was present (Figures 7(a) and 7(b)). Due to infection, IL-6 and TNF-*α* significantly increased at 24 hours. Moreover, IL-6 and TNF-*α* levels significantly increased over course of time in both, ethanol-treated mice and respective controls, indicating ongoing infection and progression of disease. Yet, alcohol pretreatment had no effect.

### 3.9. Cytokine Milieu in Plasma

**Figure 8:**
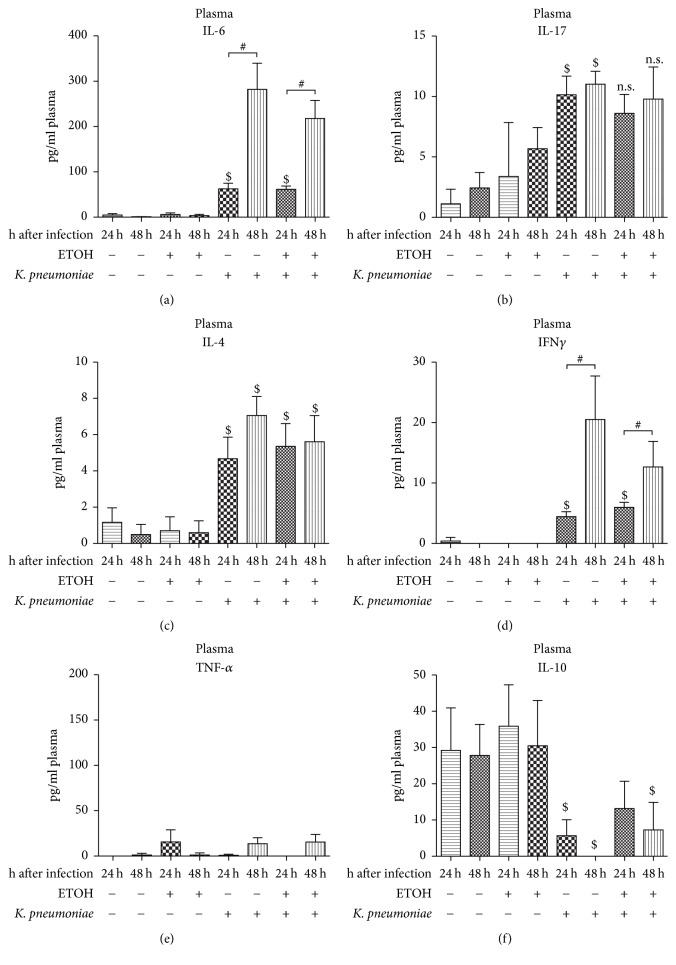
Protein levels of (a) IL-6, (b) IL-17, (c) IL-4, (d) IFN-*γ*, (e) TNF-*α*, and (f) IL-10 in plasma in a murine model of ethanol exposition for 2 weeks, surgery, and pulmonary infection. Protein levels of indicated cytokines were analyzed by cytometric bead array (CBA) and given as pg/ml plasma of ethanol-treated (ETOH) and saline-treated controls (NaCl) at 24 hours or 48 hours after pulmonary infection with* Klebsiella pneumoniae*. ^$^*p* < 0.05 when compared to time- and treatment-matched control (infection-dependent effects); ^#^*p* < 0.05 when comparing 24 hours to 48 hours (time-dependent effects); data are presented as mean ± SEM, Mann–Whitney* U* test (*n* = 8 each group). n.s.: not significant. IL: interleukin; TNF-*α*: tumor necrosis factor; IFN-*γ*: interferon gamma; ETOH: ethanol treatment;* K*.* pneumoniae*: pulmonary infection with* Klebsiella pneumoniae*.

To gain insight into the cytokine profile released into the circulation in our experimental setting, we analyzed pro- and anti-inflammatory cytokines in all experimental groups (Figures 8(a)–8(f)). The observed increase of proinflammatory IL-6 in the lung was also detectable in the blood and reached significant levels in an infection scenario (Figure 8(a)). This was also true for proinflammatory IFN*γ* (Figure 8(b)). Cytokine levels of proinflammatory IL-17 were by trend higher in ethanol-treated animals (Figure 8(c)). However, IL-17 levels were significantly higher with infection. Importantly, this effect was abolished by previous ethanol treatment. Circulating levels of TNF-*α* were low (Figure 8(e)), whereas levels of IL-4 were significantly increased whenever infection was present (Figure 8(c)). Anti-inflammatory IL-10 was overall downregulated with infection (Figure 8(f)). However, there was no statistically significant effect of alcohol pretreatment.

## 4. Discussion

Given the fact that patients with alcohol use disorder (AUD) develop a significantly higher postsurgical risk of infections, we established a murine three-hit bench to bedside model, including all three stress factors alcohol, surgery, and infection. We analyzed differentiation of T helper cell subpopulations, the release of their signature cytokines, and physical outcomes in this model using pulmonary infection in the context of a two-week history of ethanol exposure followed by surgery.

The most important finding of our study is that both, surgery and ethanol exposure, lead to subtle, but significant modulations in T helper cell subset differentiation and cytokine milieu in an infection scenario. Alterations of absolute numbers and cellular functionality of T helper cells may aggravate the pulmonary infection and thereby impair the clinical outcome.

In our model, administration of ethanol alone for two weeks did not lead to a distinct impairment of clinical condition in respective animals. Moreover, body weight which serves as a sensitive parameter for impairment of physical well-being did not differ significantly at day 14 of our experiment, that is, before experimental surgery and before pulmonary infection. This is comparable with data from our previous experiments engaging a period of ethanol exposure for 8 days [20, 23]. We found that alcohol intake prior to surgery worsened clinical appearance, led to a more severe pulmonary infection with* K. pneumoniae*, and was associated with more tissue destruction. However, in this previous work we could not detect effects that became apparent only by alcohol treatment. This means that all alcohol effects were only evident with further “hits,” that is, surgery and/or pulmonary infection. The duration of alcohol exposition is critical for its impact on immunity. Longer exposition times usually lead to a more pronounced impairment of the immune system. There is, however, no universal definition as to what constitutes an “optimal” alcohol treatment period in animal models. Typically, short-term alcohol given within a period of up to two weeks is regarded as “acute administration.” Within this time frame chronic organ complications (e.g., alcoholic liver disease or hepatitis) as they are seen after longtime alcohol treatment do not occur [24]. Yet, already a one-week history of ethanol exposition induces damage of the immune system, as shown previously [20, 23]. As expected, in our recent model ethanol treatment was associated with deterioration of physical well-being after experimental surgery as indicated by impaired clinical scoring and loss of body weight in respective animals. Consistently, loss of body weight after surgery relative to the first day of the experiment was significantly higher in alcohol-treated animals as compared to controls. Moreover, saline-treated animals exhibited a shorter recovery period. Ethanol-treated mice had a worse clinical scoring throughout the postsurgical phase, reflecting a more severe physical impairment. Both simulated conditions, that is, experimental surgery and pulmonary infection, were as expected associated with indicators of poor physical well-being and clinical condition. Ethanol aggravated the clinical impairment in an additive manner.

Intranasal administration of* K. pneumonia* led to a detectable induction of pneumonia. This is shown by a considerable amount of CFUs in lung tissue after inoculation and is consistent with histological findings described previously within the same infection model [20]. Chronic ethanol exposition is reported to substantially compromise immunity [25] and to impair pulmonary clearance of gram-negative bacteria [22]. As hypothesized, in our model the amount of CFUs in lung tissue and blood was significantly higher in ethanol-treated, infected mice as compared to respective control groups over course of time. This ethanol-induced effect, however, is in line with previous findings and indicates a more severe pulmonary and systemic state of infection in ethanol-treated mice in our experimental setting. Ethanol-induced abnormalities of adaptive immunity are known to impair pulmonary immune competence and to render the lung more susceptible to infectious complications [26]. Overall, count of CD3^+^ T cells was significantly increased in infection in both, ethanol-treated and control groups. This demonstrates responsiveness of T cell mediated immunity in our model. Classical proinflammatory cytokines of early response such as IL-6 or TNF-*α* were also significantly induced due to infection. IL-6 is a reliable marker of the magnitude of injury and corresponds with the degree of tissue damage and systemic inflammation [27, 28]. IL-6 is significantly upregulated in our model due to infection. Correspondingly, IL-10 is significantly downregulated and the IL-6/IL-10 ratio is shifted towards a more proinflammatory state.

Lung CD4^+^ IFN-*γ* producing T cells (i.e., T cells of the Th1 lineage) were unchanged at 24 hours in either ethanol-treated or control groups with and without infection in our model. However, IFN-*γ* as the signature cytokine of Th1 cells was significantly increased in infection in lungs and plasma reflecting an adequate proinflammatory response. Yet, by trend there was an inhibition of IFN-*γ* levels in plasma in ethanol-treated animals. Although this ethanol-induced effect could not be detected on the cellular level, this general observation is in line with data from the literature, since ethanol exposition has been previously shown to suppress the Th1 immune response [29]. CD4^+^ IL-4 producing T cells were significantly increased in ethanol-treated, infected mice at 24 hours, but not in respective sham-infected control group. At this early stage of infection, anti-inflammatory Th2 cells and the inhibiting cytokine IL-4 thereby outweighed proinflammatory Th1 cells and IFN-y levels in ethanol-treated groups. This effect, however, was transient but might reflect an early imbalance in Th1/Th2 ratio promoting progress of infection. In humans, a diminished Th1/Th2 ratio was shown as a consequence of ethanol abuse. This may contribute to the increased susceptibility of postsurgical, infectious complications in these patients [30]. Transferred to our model, this mechanism might have impaired host defense and may have facilitated progress of pulmonary infection.

IL-17 producing CD4^+^ T cells were significantly increased in lungs of alcohol-treated, infected mice, but not in saline-treated, infected control groups. On the other hand, there was no difference in Th17 cell count in lungs of uninfected animals with and without previous ethanol exposition at all. We did not observe significant differences of IL-17 levels in lungs of infected animals with and without ethanol exposition, even though we detected a significantly increased Th17 cell count. Thus, we conclude that functionality of proinflammatory Th17 cells might have been hampered in an ethanol-induced manner in these animals. A relative, local decrease of IL-17 in ethanol-treated mice as compared to control animals might—at least in part—be promotive for ongoing pulmonary infection. IL-17 is known to be engaged in protective immunity against various infections like* K. pneumoniae* [31]. A relative decrease of IL-17 is associated with increased mortality [32].

We did not detect any differences in either intervention with regard to Treg cells at 24 hours in our model. However, with progression of infection at 48 hours, Treg cells were significantly reduced in both, ethanol-treated and saline-treated controls, with no difference between these intervention groups. In several animal models, experimental surgery led to a substantial increase of regulatory T cells likewise in lung and spleen. This effect was also observed in other trauma models when Th1 response was significantly suppressed by Treg cells due to injury [33]. It is suggested that this may contribute to the increased susceptibility of postsurgical, infectious complications. However, the counterinflammatory properties of Treg cells might contribute to a physiological response after trauma [34]. An initial hyperinflammatory burst is followed by a compensatory anti-inflammatory response [35, 36]. In our model, on the other hand, we combine experimental surgery and infection. We suggest that downregulation of Treg cells in our setting might be a physiological response to infection. Upregulation of anti-inflammatory Treg cells in an infection scenario might hamper the proinflammatory immunity and have deleterious consequences for the host. Treg cells do not seem to be involved in alcohol-induced immune derangement in our experimental setting.

The analysis of engaged master-regulatory transcription factors of distinct Th-sublineage commitment was inconclusive. Regulatory influence of either surgery, infection, or ethanol treatment did not necessarily translate into measurable and distinctly different cell counts of T helper cell subpopulations. Moreover, the cytokine release in the lung did not correlate well with the expression of respective master transcription factors. However, T helper cell subdifferentiation is complex and regulated on multiple stages with mutual interferences. Besides, expression of transcription factors might not necessarily reflect their stage of activation. Therefore, further experimental research is necessary to elucidate the entanglement of the regarded master transcription factors in the context of infection, surgery, and ethanol exposition.

In conclusion, our murine bench to bedside model involving pulmonary infection showed that both, ethanol and surgery, led to significant and complex modulations of T helper cell subsets on both the cellular and functional level. Ethanol led to a worse clinical outcome and a more severe pulmonary infection. We suggest that these effects in addition to the classical Th1/Th2 paradigm, at least in part, may contribute to ethanol-induced immunosuppression and the impairment of the clinical course of infections. However, our model only concerns the mere effect of ethanol on the immune cells analyzed by us. It was not our intention to address the condition “alcoholism” which involves alcohol dependency and withdrawal. Whether our results hold true for human conditions and whether they provide with a mechanism of ethanol-induced immunosuppression have to be assessed in further, clinical studies.
